# Low Incidence of SARS-CoV-2 in Patients with Solid Tumours on Active Treatment: An Observational Study at a Tertiary Cancer Centre in Lombardy, Italy

**DOI:** 10.3390/cancers12092352

**Published:** 2020-08-20

**Authors:** Alexia Francesca Bertuzzi, Andrea Marrari, Nicolò Gennaro, Umberto Cariboni, Michele Ciccarelli, Laura Giordano, Vittorio Lorenzo Quagliuolo, Armando Santoro

**Affiliations:** 1 Medical Oncology and Hematology Unit, Humanitas Clinical and Research Center, IRCCS, 20089 Milan, Italy; alexia.bertuzzi@humanitas.it (A.F.B.); andrea.marrari@humanitas.it (A.M.); 2 Department of Biomedical Sciences, Humanitas University, 20072 Milan, Italy; nicolo.gennaro@st.hunimed.eu; 3 Radiology Unit, Humanitas Clinical and Research Center, IRCCS, 20089 Milan, Italy; 4 Thoracic Surgery Unit, Humanitas Clinical and Research Center, IRCCS, 20089 Milan, Italy; umberto.cariboni@humanitas.it; 5 Pneumology Unit, Humanitas Clinical and Research Center, IRCCS, 20089 Milan, Italy; michele.ciccarelli@humanitas.it; 6 Biostatistics Unit, Humanitas Clinical and Research Center, IRCCS, 20089 Milan, Italy; laura.giordano@humanitas.it; 7 Sarcoma, Melanoma and Rare Tumors Surgery Unit, Humanitas Clinical and Research Center, IRCCS, 20089 Milan, Italy; vittorio.quagliuolo@humanitas.it

**Keywords:** COVID-19, SARS-CoV-2, cancer care, chemotherapy, immunotherapy, treatment safety

## Abstract

**Background:** The incidence and prognosis of SARS-CoV-2-positive cancer patients on active oncologic treatment remain unknown. Retrospective data from China reported higher incidence and poorer outcomes with respect to the general population. We aimed to describe the real-word incidence of SARS-CoV-2 in cancer patients and the impact of oncologic therapies on the infection. **Materials & Methods:** In this study, we analysed all consecutive cancer patients with solid tumours undergoing active intravenous treatment (chemotherapy, immunotherapy, targeted therapy, alone or in combination) between 21 February and 30 April 2020, in a high-volume cancer centre in Lombardy, Italy. We focused on SARS-CoV-2-positive patients, reporting on the clinical characteristics of the cancer and the infection. **Results:** We registered 17 SARS-CoV-2-positive patients among 1267 cancer patients on active treatment, resulting in an incidence of 1.3%. The median age was 69.5 years (range 43–79). Fourteen patients (82%) required hospitalisation for COVID-19 with a median in-hospital stay of 11.5 days (range 3–58). Fourteen of the seventeen (82%) were treated for locally advanced or metastatic disease. We could not demonstrate any correlation between SARS-CoV-2 infection and tumour or treatment type. The COVID-19-related fatality rate was 29% (5/17), which was higher than that of the general population cared for in our centre (20%). **Conclusions:** Active oncologic treatments do not represent a risk factor for SARS-CoV-2 infection in cancer patients. However, the prognosis of infected cancer patients appears to be worse compared with that of the non-oncologic population. Given the low number of SARS-CoV-2-positive cases and the uncertainties in risk factors that may have an impact on the prognosis, we advocate for the continuum of cancer care even during the current pandemic.

## 1. Introduction

The coronavirus pandemic represents an international emergency with a dramatic impact on the healthcare and socioeconomic systems. At the time of writing, the spread of the outbreak accounted for more than 8 million SARS-CoV-2-positive patients and more than 450,000 deaths. Italy was the first European country where the COVID-19 outbreak took hold [1]. In particular, Lombardy has been affected considerably with more than 83,000 positive cases and 15,000 deaths. As a cancer referral centre with 6500 patients on treatment and 30,000 on follow-up per year, our institute was appointed by the local government as one of the three regional hubs for oncologic care. Moreover, being the only hub with an emergency department, it became one of the centres at the forefront of COVID-19 care. In this scenario, providing care both to COVID-19 and oncologic patients represented a unique challenge. The limited in-hospital resources, along with uncertainties regarding the safety of cancer treatments in a vulnerable population, represented the major concerns of our clinical practice.

The severe immunosuppression caused by cancer itself and oncologic treatments represents a potential threat to patients, as confirmed by initial retrospective data from China. However, limited information on tumour types, the extent of disease, or the impact of anticancer treatments on the risk of infection has been reported [2]. We focused on the oncologic population on active treatment at a single cancer centre during the most critical months of the pandemic, describing their clinical characteristics, along with the incidence and outcome of SARS-CoV-2 infection [3,4].

## 2. Materials and Methods

### 2.1. Study Design

We reviewed the medical records of all consecutive adult patients affected by solid malignancy receiving oncologic treatment in the Medical Oncology Department of a single high-volume cancer centre between 21 February and 30 April 2020. We collected data on anticancer treatments (chemotherapy, immunotherapy, and/or targeted therapy, alone or in combination), delivery settings (inpatient and outpatient), number of hospital accesses, and SARS-CoV-2 status (“positive and negative patients on treatment”). We were unable to extend our analysis to patients on hormonal therapy due to the timeline of their follow-up, as well as the hospital reorganisation and the national lockdown. Positivity for SARS-CoV-2 was defined on the basis of nasopharyngeal swab or bronchoalveolar lavage (BAL) positivity, regardless of clinical-radiological presentation (symptomatic or asymptomatic patients with suggestive imaging on CT scan). Demographic and clinical characteristics of the SARS-CoV-2-positive oncologic patients were collected and analysed. In particular, we reported on comorbidities (focusing on hypertension, coronary artery disease, diabetes, dyslipidemia, and chronic obstructive pulmonary disease, COPD), body mass index (BMI), smoking habits, tumour and treatment type, aim of the treatment (palliative vs. curative), granulocyte colony-stimulating factor (G-CSF) use, and disease status (localised/locally advanced vs. metastatic). In these patients, we also described the clinical presentation, the radiologic findings, and the outcome of SARS-CoV-2 infection and their oncologic disease. Similarly, we registered and described SARS-CoV-2-positive patients with a recent diagnosis of cancer waiting for treatment admitted in the same period (“positive, treatment-naive patients”). The absence of prospective informed consent was waived by the Ethics Committee due to the emergency situation in the clinical scenario.

### 2.2. Statistical Plan and Data Management

Due to the small sample size, no statistical test could be performed and results are explorative in nature. Data were described as frequencies and proportions or as median and range.

## 3. Results

Between 21 February and 30 April 2020, 814 inpatients had over 2540 treatment sessions with intravenous medications, including chemotherapy, immunotherapy, and targeted therapy (alone or in combination) in our Medical Oncology Department.

In the outpatient setting, 453 outpatients received oral chemotherapy or targeted therapy, for a total of 817 accesses (Table 1). Out of 1267 cancer patients (814 inpatients and 453 outpatients), we registered 17 SARS-CoV-2 “patients positive on treatment,” resulting in an overall incidence of 1.3%. The demographic and oncologic characteristics of these patients are summarised in Table 2. The median age was 69.5 years (range 43–79). Fourteen patients (14/17, 82%) had locally advanced or metastatic cancer, while the rest had no evidence of disease (3/17, 18%). Treatment was palliative in 13 patients (13/17, 76%) and potentially curative in 4 (4/17, 24%). In the latter group, three patients were on adjuvant treatment and one on neoadjuvant chemotherapy. The characteristics of SARS-CoV-2 infection are summarised in Table 3. Most of the patients were hospitalised for COVID-19 (14/17, 82%), whereas the rest were addressed to self-isolation (3/17, 18%).

The symptoms at presentation were fever (9/17, 53%), shortness of breath (4/17, 23%), abdominal pain (2/17, 12%), and shock (2/17, 12%). The median time from the onset of symptoms to hospital admission was 3.5 days (range 0–8). Treatment consisted of hydroxychloroquine (11/17, 65%), antivirals (7/17, 41%), antibiotics (7/17, 41%), and oxygen therapy (8/17, 47%). The median hospital stay for the treatment of COVID-19 was 11.5 days (range 3–58). None of the patients were admitted to the intensive care unit (ICU). The overall COVID-19-related fatality rate among the oncologic patients on active treatment was 29% (5/17), while among the hospitalised patients, it rose to 36% (5/14). Among the group of SARS-CoV-2-positive “patients treatment-naive,” we identified nine patients admitted to the hospital with a recent diagnosis of cancer. The clinical characteristics of cancer and SARS-CoV-2 infection for these patients are summarised in Table 4. The median age was 79 (range 63–89). All but one patient had advanced disease, four metastatic, and four locally advanced nonresectable tumours. Six patients had concomitant cardiovascular diseases (6/9, 67%). The mortality rate among this group was 78% (7/9).

## 4. Discussion

Oncologic patients represent a population at risk of developing infections due to the immune suppression caused by cancer itself and treatments. The SARS-CoV-2 outbreak suddenly became a serious threat to such a vulnerable population, where the scientific community was expecting an uncontrolled spread of the infection [2,6,7]. Early data from China seemed to confirm these fears, reporting a high incidence of cancer patients among patients hospitalised for COVID-19, up to 6% [2,7,8,9,10]. Similarly, initial experiences from the ICUs in Lombardy reported that 8% of all the admitted patients were oncologic [11]. In this uncertain scenario, national and international guidelines promptly recommended extreme caution when prescribing oncologic therapies [12,13]. Still, in the aforementioned studies, no information regarding the extent and/or status of the disease at the time of the infection, as well as the ongoing oncologic treatments, was detailed [2,6,7]. Moreover, all these papers report the incidence of cancer patients among all the patients admitted for COVID-19.

To the best of our knowledge, this analysis describes for the first time the real-world incidence of SARS-CoV-2 infection in a cancer population on active medical treatment. We did not confirm a high risk for SARS-CoV-2 in patients treated with chemotherapy, immunotherapy, or targeted therapy, as we identified only 17 SARS-CoV-2-positive patients out of 1267 consecutive cancer patients, resulting in an incidence of 1.3%. Although the low number and heterogeneity of cases hampered any statistical analysis, we observed that most SARS-CoV-2 cancer patients presented with advanced disease or were on palliative treatment. No correlation between tumour type and treatment, as well as treatment settings, could be identified. Among our patients, treatment-induced febrile neutropenia requiring G-CSF was recorded only in two patients, questioning the role of neutropenia as a risk factor for SARS-CoV-2 infection. However, the recommended use of G-CSF in this scenario should be carefully evaluated due to its potential involvement in the cytokine release storm observed in infected patients.

Overall, the characteristics of the infection in cancer patients on treatment were similar to those in the non-oncologic population in terms of age, comorbidity, and clinical presentation [3]. It is noteworthy that we did not observe any positive cases in patients under 40 years of age, despite our institute being a high-volume adolescent and young-adult cancer centre. Regarding the fatality rate of COVID-19, we registered a higher percentage compared with that in the entire population admitted in our institute (29% and 20%, respectively) [3]. Published data on the mortality rate of COVID-19 in these patients are conflicting. The UK Coronavirus Cancer Monitoring Project (UKCCMP) reported it to be 28% in patients with haematological and solid tumours (226/800), which slightly decreased to 25% (101/397) when evaluating only those patients on treatment within four weeks from the diagnosis of COVID-19. The authors concluded that age, gender, and comorbidity, unlike cancer therapy, are major risk factors [14]. Conversely, the Chinese experience highlighted a fatality rate of 20% (40/205), without specifying how many patients did receive therapies within four weeks from the onset of symptoms. Chemotherapy was identified as a significant unfavourable risk factor for mortality, suggesting that long-lasting myelosuppression and impaired immunity may play a negative role in the clinical outcome of such patients [15]. No information on G-CSF use was reported, precluding any correlation between clinical outcomes and the use of these agents in SARS-CoV-2-infected patients.

The high fatality rate we observed in hospitalised cancer patients could be a result of several factors, including the burden of disease and/or the status of malignancy, as well as the reported comorbidities. Unfortunately, we were unable to conduct a multivariate analysis, which is mandatory to establish the real value of each risk factor for SARS-CoV-2. In particular, all patients on adjuvant treatment recovered from the infection without sequelae. Unfortunately, no information on the status of the disease at the time of SARS-CoV-2 infection was available for patients with advanced disease, thus making any correlation between cancer response and the prognosis of the infection unfeasible. To strengthen this hypothesis, we highlight the high fatality rate (7/9, 78%) we registered in the subgroup of “treatment-naive patients” we admitted during the first weeks of the pandemic and characterised by uncontrolled tumour growth.

We acknowledge that our study has several limitations. The limited number of positive cases did not allow us to identify any significant association of SARS-CoV-2 infection with specific treatments, tumour types, and settings. Moreover, the observational nature of the study exposed our analysis to selection biases due to the modality used in the identification of cases and the collection of clinical data. Moreover, as for the general population in Italy, our data might underestimate the incidence of SARS-CoV-2, as asymptomatic cancer patients undergoing medical therapy in the outpatient setting were not routinely screened for SARS-CoV-2. Given the limited sensitivity of the available diagnostic tests (71–98%) [16], we may have missed patients with suggestive imaging on chest CT scan but negative nasopharyngeal swab or BAL. Despite the study design, the tight treatment schedules and the close patient monitoring characterising our cancer centre guaranteed an accurate analysis of oncologic patients on active treatment during the COVID-19 outbreak.

In conclusion, our data indicate that anticancer treatments do not represent a risk factor for SARS-CoV-2 infection, thus questioning the fears of the medical oncology community of treating this supposedly vulnerable population. COVID-19-related mortality could be due to a combination of several risk factors other than cancer itself. We are convinced that the major risk for cancer patients is still represented by the delay or lack of treatment. This awareness should support cancer centres to maintain their oncologic commitments even under extraordinary circumstances like the current COVID-19 pandemic.

## Figures and Tables

**Table 1:** Distribution of SARS-CoV-2 infection among cancer patients according to treatment and delivery setting.

	Chemo	Immuno	Targeted	Chemo-Immuno	Chemo-Targeted	Hormonal-Targeted	Other	Total (%)
Inpatients	405	129	128	19	98	0	35	814
SARS-CoV-2	5	2	0	1	1	0	1	10 (1.2%)
Outpatients	194	0	182	0	0	77	0	453
SARS-CoV-2	1	0	4	0	0	2	0	7 (1.5%)

**Table 2:** Clinical characteristics of SARS-CoV-2-positive cancer patients on active treatment.

Patient	Sex	Age	Cancer Diagnosis	BMI	Smoking	Comorbidities	Stage of Disease	Active Treatment	G-CSF
**1**	M	77	Prostate cancer	29.7	Yes	HT, DLD	Metastatic	Chemo-Immuno	Yes
**2**	F	65	Breast cancer	25.3	No	HT, DLD	Metastatic	Hormonal-Targeted	No
**3**	M	71	Laryngeal small cell cancer	27.6	Yes	HT, DLD, CAD, DM	Locally advanced	Chemo	Yes
**4**	M	55	CNS	23.0	No	-	Local recurrence	Chemo	No
**5**	F	58	Renal clear cell cancer	21.8	No	HT	Metastatic	Targeted	No
**6**	M	43	CNS	27.4	No	DLD	Local recurrence	Targeted	No
**7**	F	66	Mesothelioma	23.3	No	HT	Metastatic	Chemo	No
**8**	F	79	Renal clear cell cancer	20.7	Ex	HT, RA	Metastatic	Immuno	No
**9**	M	70	Pancreatic cancer	20.0	No	CAD, DM, CVA	Adjuvant	Chemo	No
**10**	M	60	Prostate cancer	27.7	No	-	Metastatic	Immuno	No
**11**	F	45	Melanoma	33.6	No	-	Adjuvant	Targeted	No
**12**	F	79	Breast cancer	21.4	No	DLD	Adjuvant	Chemo-Targeted	No
**13**	F	71	Breast cancer	32.4	Ex	HT, COPD, RA	Metastatic	Hormonal-Targeted	No
**14**	F	60	Lung cancer	22.3	Ex	-	Metastatic	Immuno-Targeted	No
**15**	M	75	Lung cancer	35.3	Yes	HT, CAD, DLD, COPD	Metastatic	Chemo	No
**16**	F	68	Lung cancer	24.1	No	HT, PE	Metastatic	Targeted	No
**17**	M	47	Gastric cancer	25.1	No	DLD	Metastatic	Chemo	No

CNS, central nervous system; DM, diabetes; RA, rheumatoid arthritis; COPD, chronic obstructive pulmonary disease; G-CSF, granulocyte colony-stimulating factor; HT, hypertension; DLD, dyslipidemia; CAD, coronary artery disease; CVA, cerebral vascular accident; PE, pulmonary embolism.

**Table 3:** Characteristics of SARS-CoV-2 infection in cancer patients on active treatment.

Patient	Time to Diagnosis (Days)	Symptoms	IL-6 (pg/mL) *	CT Findings (SS)	Therapies	Management	In-Hospital Stay (Days)	Time to Negative Swab (Days)	Outcome
**1**	6	Abdominal pain	4	Unilateral single GGO (1)	HCQ, AV, AB, O_2_	Hospitalised	15	n/a	DOC
**2**	2	Fever, cough, SOB	n/a	n/a	HCQ, O_2_	Hospitalised	5	n/a	DOC
**3**	1	Shock	n/a	Diffuse bilateral GGO (9)	-	Hospitalised	7	n/a	DOC
**4**	2	Fever, cough	5	Bilateral basal dysventilation (0)	HCQ, AV, AB, O_2_	Hospitalised	29	n/a	Alive
**5**	0	-	3	Bilateral GGO, hilar bilateral adenopathies (2)	-	Hospitalised	3	24	Alive
**6**	0	SOB, shock	n/a	n/a	O_2_	Hospitalised	4	n/a	DOD
**7**	3	SOB	n/a	n/a	HCQ, AV, AB, O_2_	Hospitalised	30	n/a	DOD/DOC
**8**	4	Fever	n/a	Unremarkable (0)	HCQ, AV, AB, O_2_	Hospitalised	8	19	Alive
**9**	1	Fever	n/a	n/a	HCQ, O_2_	Hospitalised	30	30	Alive
**10**	8	Fever, cough	n/a	Unremarkable (0)	HCQ	Isolation	-	10	Alive
**11**	0	-	n/a	Unilateral GGO (3)	-	Isolation	-	33	Alive
**12**	1	Fever	n/a	n/a	HCQ, AV, AB, O_2_	Hospitalised	24	18	Alive
**13**	1	Fever	n/a	Unremarkable (0)	-	Isolation	-	41	Alive
**14**	1	Pain	55	GGO, pleural effusion (3)	HCQ	Hospitalised	30	14	Alive
**15**	1	Fever, SOB		GGO, hilar bilateral adenopathies (7)	HCQ, AV, O_2_	Hospitalised	58	82	Alive
**16**	1	Fever		Unremarkable (0)	HCQ, AB, O_2_	Hospitalised	8	15	Alive
**17**	1	Abdominal pain	2	Bilateral GGO (2)	HCQ, AV, AB, O_2_	Hospitalised	8	42	Alive

SOB, shortness of breath; HCQ, hydroxychloroquine; AV, antivirals; AB, antibiotics; O_2_, oxygen therapy; SS, CT severity score (0–5 points for each pulmonary lobe involved, max 25 points) [5]; DOC, dead of COVID-19; DOD, dead of disease; n/a, not available; GGO, ground-glass opacification; * normal IL-6 values < 6.4 pg/mL.

**Table 4:** Clinical characteristics of COVID-19 patients recently diagnosed with cancer (treatment-naive).

Patient	Sex	Age (Years)	Diagnosis	BMI	Smoking	IL-6 (pg/mL) *	CT Findings (SS)	Comorbidities	Stage of Disease	Management	Outcome
**1**	M	89	Bladder cancer	29.4	Ex	n/a	Bilateral ground-glass consolidation, adenopathies, pleural effusion (12)	CAD, COPD	Localised	Hospitalised	DOC
**2**	M	63	Cholangiocarcinoma	46.9	Ex	1	Bilateral diffuse ground-glass consolidation (7)	DM, HT, COPD, CAD	Locally advanced	Hospitalised	DOD
**3**	F	70	Cholangiocarcinoma	29.4	No	7	n/a	-	Locally advanced	Hospitalised	Alive
**4**	F	76	Cholangiocarcinoma	18.5	No	n/a	Left lobe consolidation, pleural effusion (5)	-	Locally advanced	Hospitalised	DOD
**5**	M	66	Lung cancer	22.6	Ex	n/a	Bilateral ground-glass consolidation, pleural effusion (6)	CAD	Metastatic	Hospitalised	DOC/DOD
**6**	F	79	Pancreatic cancer	27.6	Yes	2	Bilateral ground-glass consolidation, adenopathy, PE (20)	DM	Metastatic	Hospitalised	DOD
**7**	M	68	Oesophageal cancer	18.8	Ex	4	Bilateral ground-glass consolidation (2)	DM, HT	Locally advanced	Hospitalised	Alive
**8**	M	67	Lung cancer	23.9	Ex	7	n/a	-	Metastatic	Hospitalised	DOC/ DOD
**9**	F	69	Lung cancer	25.4	No	n/a	Bilateral ground-glass consolidation, adenopathy, pleural effusion (14)	-	Metastatic	Hospitalised	DOC/ DOD

DOC, dead of COVID-19; DOD, dead of disease; HT, hypertension; DLD, dyslipidemia; CAD, coronary artery disease; CVA, cerebral vascular accident; PE, pulmonary embolism; SS, CT severity score (0–5 points for each pulmonary lobe involved, max 25 points) [5]; * normal IL-6 values < 6.4 pg/mL.
